# Among equity and dignity: an argument-based review of European ethical guidelines under COVID-19

**DOI:** 10.1186/s12910-021-00603-9

**Published:** 2021-03-31

**Authors:** Marta Perin, Ludovica De Panfilis

**Affiliations:** 1 Bioethics Unit, Azienda USL-IRCCS di Reggio Emilia, Reggio Emilia, Italy; 2 PhD Program in Clinical and Experimental Medicine, University of Modena and Reggio Emilia, Modena, Italy

**Keywords:** COVID-19 pandemic, Allocation of Health Care Resources, Human Dignity, Ethics

## Abstract

**Background:**

Under COVID-19 pandemic, many organizations developed guidelines to deal with the ethical aspects of resources allocation. This study describes the results of an argument-based review of ethical guidelines developed at the European level. It aims to increase knowledge and awareness about the moral relevance of the outbreak, especially as regards the balance of equity and dignity in clinical practice and patient’s care.

**Method:**

According to the argument-based review framework, we started our research from the following two questions: what are the ethical principles adopted by the ethical guidelines produced at the beginning of the COVID-19 outbreak related to resource allocation? And what are the practical consequences in terms of 'priority' of access, access criteria, management of the decision-making process and patient care?

**Results:**

Twenty-two ethical guidelines met our inclusion criteria and the results of our analysis are organized into 4 ethical concepts and related arguments: the equity principle and emerging ethical theories; triage criteria; respecting patient’s dignity, and decision making and quality of care.

**Conclusion:**

Further studies can investigate the practical consequences of the application of the guidelines described, in terms of quality of care and health care professionals’ moral distress.

**Supplementary Information:**

The online version contains supplementary material available at 10.1186/s12910-021-00603-9.

## Background

After the spread of the novel severe acute respiratory syndrome coronavirus 2 (SARS-CoV-2) and the related COronaVIrus Disease 2019 (COVID-19), many national health care systems had to deal with a dramatic re-organization of services. At the beginning of the outbreak, COVID-19 positive patients with severe illness required highly specialized care, such as mechanical ventilation and extracorporeal membrane oxygenation, which were not readily available or not applicable to a significant number of cases [1–3]. Moreover, the scarce availability of intensive care unit (ICU) beds is common all over the word [4]. It forces health care professionals and health care organizations to continuously balance the principle of patient-centered care, the focus of clinical ethics under normal conditions, and that of public-focused duty to promote equality of persons, which is the focus of public health ethics [5]. More than usual, the spread of Covid-19 highlighted this difficult balancing.

In a short time, the hardest-hit countries, China, USA, Italy and Spain [3, 4] had to deal with issues of allocation of scarce resource. Rationing intensive care beds or specialized services has clinical and ethical implications. The “decision about initiating or terminating mechanical intervention is often truly a life-or-death choice” [7], even if, in the case of Covid-19, data have shown uncertainty regarding the efficacy of mechanical ventilation as the outbreak has progressed [8]. However, the allocation of intensive care treatments remains crucial.

The central question regards how to establish a fair resource allocation and how to guarantee that the decisions are made “ethically and consistently, rather than based on individual institution’s approaches or a clinician's institution in the heat of the moment” [9].

Resource allocation is a crucial ethical issue because it most fundamentally involves questions of justice. The identification and definition of an equal distribution of resources, opportunities and outcomes among the population represent a concern for national governments and health care facilities. Due to the nature of the pandemic itself, this ethical issue reached a global dimension.

According to the World Health Organization (WHO) [9], developing guidelines on the allocation of scarce resources in outbreak situations require governments, health-care facilities and other involved in such effort to consider the tension between the principles of utility (allocating resources to maximize benefit and minimize burdens) and equity (fair distribution of benefit and burdens); the definition of utility; the needs of vulnerable populations; the fulfillment of reciprocity-based obligations and the provision of supportive and palliative care. The principle of equity in access to health care plays a central role in fair allocation: it “must be upheld, because it lays down in the respect for human dignity and human right framework” (namely, Article 3 of the Oviedo Convention and article 14 of the Unesco Declaration on Bioethics and Human rights, 2005) even in a context of scarce resources [10, 11].

There are multiple ethically permissible approaches to allocating scarce life-sustaining resources, but these theories yield conflicting definitions. For instance, utilitarianism focuses on the maximization of benefit, while egalitarianism focuses on equality of opportunity and need [12].

With COVID-19, health departments, local and national clinical ethics committees, and scientific societies had urgently developed new (or have revised existing) ethical guidelines or ethical recommendations, identifying which criteria should be used to ethically allocate scarce resources and what process should be used to fairly implement allocation decisions [13].

Because the public will bear the consequences of these decisions, knowledge of perspectives and moral points of reference on these issues is critical [14]: unjustified variation could exacerbate structural inequalities, squander valuable resources and undermine public health [13, 14], as demonstrated by the debates about exclusion criteria and discrimination against age and disability that have already arisen in Italy and in the USA [15].

It can be useful to understand and compare the various ethical perspectives involved in ethical guidelines developed during COVID-19 through a full set of published reasons for or against the view in question, which is the central scope of an argument-based review [16].

This study describes the results of an argument-based review of the ethical guidelines developed at the European level to face the health care emergency at the beginning of COVID-19 pandemic.

We analyzed the underpinnings and ethical concepts, as well as their practical consequences, with the aim of increasing knowledge and awareness related to the ethical meanings of the outbreak.

## Methods

The argument-based review represents one of the different methods for systematic reviews developed in the field of bioethics, and it aims to present an up-to-date comprehensive overview of the ethical arguments and underpinning concepts identified in relation to a certain topic [16–18]. This approach is particularly suitable for our study because it led the researchers to acquire evidence for decision-making in the delivery of healthcare, development of policy, and conduct of medical research [18].

To grasp the underpinnings of ethical theories, their principles and related practical consequences regarding the ethical aspect of allocation resources, we performed the argument-based systematic review of ethical guidelines following the model developed by McCullough et al. [16, 17].

We first articulated our research questions into two relevant conceptual ethical questions; then, we performed a literature search, and finally, we identified, described and analyzed the ethical arguments in connection with the conceptual-ethical questions.

### Research questions

To understand how European countries dealt with the ethical allocation of scarce health resources at the beginning of the COVID-19 pandemic, we developed the following research questions:

- What are the ethical principles adopted by the ethical guidelines produced at the beginning of COVID-19 outbreak, related to resource allocation?
- What are the practical consequences in terms of 'priority' of access, access criteria, management of the decision-making process and patient care?

### Literature search

Articles were selected based on the following predetermined eligibility criteria for their relevance to our research question:

- they must be guidelines or recommendation policies;
- they must be published between March and May 2020;
- they must be developed in response to the COVID-19 health emergency;
- they have been developed by institutions suitable to formulate such guidelines (such as local or National Ethical Committees, Health Ministerial Departments, and Scientific Societies);
- they explicitly deal with the allocation of resource according to an ethical framework;
- they have been written in English or have been translated in English.

We excluded scientific articles, editorials, book chapters and position papers. Finally, we included further publications using the snowball sampling method and gray literature research. Due to the characteristics of the search literature, we used the following resources: national ethics committee websites, scientific society websites, international organization’s websites, collecting specific ethical resources on COVID-19 (see Additional file 1—Appendix 1).

### Data extraction and synthesis

We performed a qualitative analysis, adapting the Qualitative Analysis Guide of Leuven—QUAGOL [19]. It offers a comprehensive, systematic but not rigid method to guide the process of qualitative data analysis. Its characteristics lie in the iterative process of digging deeper, constantly moving between the various stages of the process [19].

MP and LDP identified the eligible ethical guidelines. They read each document entirely the first time to have an overview of the topics and relevant arguments; afterwards, they read the documents a second time, focusing specifically on: (a) the ethical framework described and (b) the section describing the allocation of scarce resources.

MP collected the relevant information of each document into a conceptual scheme. It is a synthetic frame where different and relevant concepts are presented and integrated with each others to answer the research questions [18, 19]. Each conceptual scheme highlights the relationships between the ethical framework described in the guideline, the allocation resources argumentation and their practical consequences in clinical practice and decision making process (an example of a conceptual scheme is provided as Additional file 2: Appendix 2). The schemes’ adequateness was checked by LDP.

Successively, MP unified all the schemes into a single table (Table 1) to create an overview and provide a comprehensive answer to our research questions. LDP overviewed the process. The table describes the responses to our research questions reporting the ethical principles (question 1) and practical consequences (question 2). We specified the relationships between ethical principles and practical consequences in terms of 'priority' of access, access criteria, management of the decision-making process and the emerging patient’s care (question 2).

**Table 1:** Ethical guidelines’ argument-based analysis: the final conceptual scheme

Country	Guidelines’ Title	Ethical principles (question 1)	Priority of access (question 2)	Access criteria (question 2)	Un-ethical access ciriteria (question 2)	Decision making process (question2)	Patient’s care approach (question 2)
Austria	Management of scarce resource in healthcare in the context of the COVID-19 pandemic	4 ethical principles; Equity equality	Better prognosis	Chronic short-term diseases Survival probability Severity of the disease Status of other previous pathologies Physical conditions Score system	Age Social status Personal relationships with decision makers	Ethics support service/ethical consultation services	Promoting Advance care planning (ACP) Reducing to the minimum the damages/sufferings resulting from the treatment, both for the patient and for the staff Offering the best, though not optimal, care for the patient and palliative care when is not possible to treat Apply a fair decision making Assistance to all people without distinctions based on non-medical criteria Provide those who need it with more resources to be able to exercise their rights (e.g. physical or mental/cognitive impairment)
Belgium	Ethical principles concerning proportionality of critical care during the COVID-19 pandemic: advice by the Belgian Society of IC medicine	Avoid disproportionate treatment apply the triage criteria fairly	Priority to urgencies Apply ‘first come, first served’ approach to those with the same urgency	Patient’s Advance Directives Presence of fragility/comorbidity Clinical Frailty Score Cognitive disorders in elderly patients Terminal onchological diseases Presence of severe chronic co-morbidities	Age	Team discussion Transparency and evaluation of decisions (using a triage decisions register) Psychological and ethical support for professionals	Promoting ACP Applying triage criteria to all patients Consider age with other clinical parameters (fragility and cognitive ability)
Council of Europe	COMMITTEE ON BIOETHICS (DH-BIO) DH-BIO Statement on human rights considerations relevant to the COVID-19 pandemic	Respect for human dignity and human right Apply the principle of equity of access to health care system Considering human right in the field of medicine (Oviedo Convention) Solidarity and responsibility		Medical criteria	Protection of the most vulnerable people (persons with disabilities, older persons, refugees and migrants)		The access to existing resources should be guided by medical criteria, to ensure namely that vulnerabilities do not lead to discrimination in the access to healthcare
Estonia	Recommendations on clinical ethics for Estonian hospitals for distribution of limited health care resources during the COVID-19 pandemic	Equal treatment 4 principles of medical ethics Avoid the greater damage and promote the maximum benefit honest and transparent distribution of limited resources	Prognosis regarding the treatment’s success The patient’s future quality of life	The current patient’s clinical status Presence of comorbidities The general patient’s health-related status Presence of other relevant indicators related to prognosis Patient’s will effectiveness of medical services	Age Gender Ethnicity Social status	Additional resources (psychologists, consultants..)	Treat all patients equally Save as many lives as possible equal distribution of existing resources ensure that the protection of health workers becomes increasingly essential
EGE	Statement on European Solidarity and the Protection of Fundamental Rights in the COVID-19 Pandemic						Derogations of human rights, albeit in the interests of the public good, must be temporary, and critically. There must be clear, transparent criteria
France	COVID-19 Contribution from the French National Consultative Ethics Committee. Ethical issues in the face of a pandemic	Respect for the dignity of the person Principle of equity	definition of priorities requires criteria which are always questionable			Unit of ethics support for health care professionals	Provide assistance based on the patient's needs Guarantee continuity of care for other patients who do not accede to intensive treatments
Germany	Solidarity and Responsibility during the Coronavirus Crisis	Dignity Absolute value of life Constitutional principles	The law does not identify any criteria by which to identify the patients to be denied the treatment		Gender Ethnicity Age Social role, value or presumed life-span	Considerations regarding allocation resources should be weighted, justified, transparent, and criteria should be applied uniformly	Equal access for all to health care The state must refrain from norms with which lives are categorized on the basis of gender/ethnicity/age/social role/presumed value or duration of life; The measures required to save as many lives as possible must not go beyond the constitutional framework and the safeguard of the legal system must be considered.
Ireland	Ethical Framework for Decision-Making in a Pandemic	Fairness, minimising harm, solidarity and reciprocity	Patients with a greater chance of benefitting from the intervention; Some groups at risk and those essential for the management of a pandemic	Patient’s health status before the virus Patient’s will Presence of comorbidities Frailty (regardless of age) Estimation on total number of lives saved; total life years saved how long patients could live in the long term	Age Social status Social value Ethnicity Gender	Reasonableness Openness and Transparency Incusiveness Responsiveness Accountability	Maximize the benefits obtained with scarce resources Distribute benefits and risks equally through a multi-principled approach
Italy	Clinical Ethics Reccomendations for the Allocation of Intensive Care Treatments in Exceptiona, resource-limited circumstances	Clinical appropriateness and Proportionality of care; Distributive justice and Appropriate allocation of resources	Age threshold: priority for those patients who are most likely to survive and who’ll have several years of life saved	Presence of comorbidities Evaluation of patient’s functional status Presence of patient’s Advance Directives or Advance Care Planning ‘Inappropriateness’ is justified by the extraordinary nature of the situation		Shared decision making process among multiple clinicians Use a ideal list of patients Daily reassessment of appropriateness/care objectives/proportionality Support to health care professionals	Maximizing benefits for most people Palliative care (also sedation) Evaluation of the situation’s implications on family members
Republic of San Marino	Statement on ethical issues regarding to the use of invasive assisted ventilation in patient s all age with serious disabilities in relation to Covid-19 pandemic	Respect for human dignity and human rights Equality and non-discrimination (due to disability) Equal opportunities to access	Clinical appropriateness Proportionality of care		Age, Gender Social status, ethnicity, disability		The only parameter for the allocation decisions consists in a correct application of the triage which is based on: (a) the respect for every human life (b) criteria of clinical appropriateness and proportionality of the treatments
Portugal	CNECV statement: Covid-19 key consideration	Value of life, dignity and integrity of individuals Principle of necessity Principle of solidarity		The evaluation of the clinical criteria and the technical and scientific recommendations must be accompanied by a careful ethical reflection based on the case studies		Permanent support from the members of the local ethics committees to help professionals in the decision-making process	Protect the health of each citizen Mitigate asymmetries and inequalities
	Public health emergency situation due to the COVID-19 pandemic - Relevant ethical aspects	Principle of necessity Precautionary principle Proportionality principle Transparency Solidarity Subsidiarity		Medical criteria Evaluation of the respective clinical criteria, including the technical and scientific recommendations issued by the health authorities, professional bodies and scientific societies		Support for decision-making through members of the health institution not directly involved in intensive care (hospital ethics committees) Principle of decision-making process: reasonableness, transparency, inclusion, reactivity and institutional responsibility	Decisions regarding the allocation resources are based on medical criteria which is based on solid ethical principles (proportionality, reciprocity, equity, trust and solidarity); careful ethical consideration is required on a case-by-case basis
Spain	Report of the Ministry of Health on ethical aspects in pandemic situations: SARS-CoV-2	Equity and non discrimination Solidarity Justice Proportionality Transparency	A hierarchy of priorities must be established Gravity of the patient's condition Objective expectations on the patient’s short-term recovery to his previous state of health Date of arrival (not as the only criterion)	Existence or absence of serious concomitant pathologies that would indicate a fatal prognosis (such as a terminal disease with a prognosis of irreversibility or irreversible coma), even if this could lead to further clinical assistance	Age Disability Vulnerable children	It is recommended that guidelines are requested and received, for example, by the hospital's ethics and health committee, within the time available	Priorities’definition will be based on objective, generalizable, transparent, public and consensus-based criteria, despite the possibility of evaluating the unique and individual characteristics of each person who has contracted the virus The maximum benefit in saving lives, which must be made compatible with the continuation of the treatment started with each individual patient) Consider alternative treatments to invasive mechanical ventilation provided in intensive care, even in cases where this does not seem to be indicated
Switzerland	Pandemic Covid-19: triage of intensive care treatments in case of scarcity of resources Indications for the implementation of chapter 9.3 of the directives of the ASSM "Measures of intensive care" (2013), updated version of March 24, 2020	4 Principles of medical ethics equity Save as many lifes as possible Protection of the health care professionals involved	Patients who can benefit most from the hospitalization It also indirectly includes the patient’s age (even if is not considered as a valid criterion itself)		The patient's age ≪ first principle such as, first served ≫ , priority to people with A high social value etc		Early identification of patients' wishes If ICU treatments are denied, adequate palliative care must be ensured Determining criterion for triage and short-term prognosis Further criteria such as first come, first served and, priority to persons with A high social value etc. should be avoided
The Holy See	Pandemic and universal Brotherhood: Note on the Covid-19 emergency	Equal value of human life and the dignity of the person (they are always the same and priceless) Justice	Patient’s need patient’s prognosis	The severity of patient’s illness and his need for treatment The evaluation of the clinical benefits obtained by the treatment, in terms of prognosis	Age cannot be taken as a single and automatic choice criterion	The allocation criteria should be shared and reasonably founded, to avoid arbitrariness or improvisation in emergency situations	Provide treatments in the best possible way based on the patient's needs The sick person should be never abandoned, even when there are no more treatments available: palliative care, pain treatment and accompaniment should never be overlooked
UNESCO International Bioethics Committee (IBC)and theUNESCO World Commission on the Ethics of Scientific Knowledge and Technology (COMEST)	Statement on COVID-19: Ethical considerations from a global perspective	Principle of justice, beneficence Equity Respect for human dignity Human rights framework recognize the protection of health as a right of each human being	right to health can be guaranteed only by our duty to health		Recognition of a collective responsibilities for the protection of vulnerable persons and the need to avoid any form of stigmatization and discrimination	Procedures need to be transparent and should respect human dignity	The highest attainable standard of health is a fundamental right of every human being, which means the access to the highest available healthcare
UK	Guidance: Responding to COVID-19: the ethical framework for adult social care	Respect Reasonabless Minimising harm Inclusiveness Flexibility Proportionality Communty				Justification of the decision-making process, considering alternative courses of action, clear, transparent decision-making process Be transparent about why certain decisions are made	Patient’s informed consent Minimize inequalities
	Ethical considerations in responding to the COVID-19 pandemic	Proportionality, Interventon’s effectiveness and necessity Fair and respectful treatment Solidariety	All the people should be treated as moral equals, worthy of respect				Interventions should be evidence-based and proportionate People should be treated as moral equals, worthy of respect
	COVID-19—ethical issues. A guidance note	maximising the overall reduction of mortality and morbidity Need to maintain vital social functions	Clinically relevant elements about each patient Patient’s possibilities to benefiting From available resources (younger patients will not automatically have priority over older ones)	The presence of comorbidities Decisions regarding treatments of those who lack decision-making capacity should be made in the same way as all the others Patients requiring treatment It would not be ethical apply these limits in health care access differently to patients with or without appointed or surrogate decision makers, or those with or without particular religious opinions		The decision making process should be based on the best available clinical data and opinions; consistent with ethical principles and reasoning Agreed in advance where possible, while recognizing that decisions may need to be made quickly Revised in changing circumstances as far as possible coherent between Different professionals Communicated openly and transparently Subjected to change and review as the situation develops Provide adequate support, including support from the clinical ethics committee and psychologists to health care professionals	Prioritisation policies: Refuse someone potentially life-saving treatment where someone else is expected to benefit more from the available treatment No automatic priority Patients whose treatment is suspended or withdrawn must receive compassionate care and dedicated medical assistance
	Ethical dimension of COVID-19 for front-line staff	Ensuring fair and equitable care Caring for COVID-19 and non-COVID-19 patients	There will be some patients (with or without confirmed COVID-19) for whom admission to ICU would be inappropriate (proportionality)			Assessment and prioritisation decisions are carried out by more than one clinician colleague (multidisciplinary team)	Treatment should be provided, independently of the individual’s background (e.g. disability), where it is considered that it will help the patient survive and not harm their long-term health and wellbeing. Many front-line staff will already be caring for patients for whom any escalation of care, regardless of the current pandemic, would be inappropriate, and must be properly managed. All front-line staff should have discussions with those relevant patients for whom an advance care plan is appropriate
	Covid-19 Guidance: Ethical Advice and Support Framework	Respect Fairness Minimising harm Working toghether Flexibility Reciprocity Capacity and consent	Where there is a decision that a treatment is not clinically appropriate there is not an obligation to provide it No active steps should be taken to shorten or end the life of an individual, however the appropriate clinical decision may be to withdraw life prolonging or life sustaining treatment			Clinicians should act with honesty and integrity in their communication with patients and should communicate clinical decisions and the reasoning behind them transparently. This should be documented appropriately Ethical advice and support groups will be established as a priority. There must be immediate access to ethical advice if this occurs, to offer an independent view and support in difficult circumstances	All patients should be offered good quality and compassionate care Patients should be treated as individuals, and not discriminated. Where there are resource constraints, patients should receive the best care possible, while recognising that there may be a competing obligation to the wider population
	Coronavirus: Your frequently asked questions	React responsibly and reasonably to the circumstances	Take account of current local and national policies that set out agreed criteria for access to treatment Take account of patient wishes and expectations	Be confident that decisions are based on clinical need and the likely effectiveness of treatments	Don’t unfairly discriminate against particular groups	Be open and honest with patients and the rest of the healthcare team about the decision-making process and the criteria for setting priorities in individual cases. Keep a record Discussion with colleagues and, if possible, with input from local ethics committees. Recognise the significant emotional distress	Provide the best service possible within the resources available. Where decisions are made to withhold or withdraw some forms of treatment from patients, doctors should still take all possible steps to alleviate the patient’s symptoms and distress and respect their dignity

The documents’ full texts, their relative conceptual schemes, and the final table were iteratively evaluated and checked against previous QUAGOL steps to ensure that they were consistent.

Finally, MP and LDP synthesized a description of the results to be presented in the Results section.

## Results

We collected 42 ethical guidelines, and their characteristics are described in Table 2.

**Table 2:** Published guidelines characteristics

Characteristics	Number of published ethical guidelines
*Country*	
Austria	1
Belgium	1
Estonia	1
France	6
Finland	1
Germany	3
Greece	1
Ireland	1
Italy	2
Luxembourg	1
Norway	1
R. of San Marino	1
Portugal	2
slovenia	1
Spain	4
Sweden	1
Switzerland	3
The Holy See	1
UK	7
International organization	3
*Published by*	
Scientific Society (professionals)	21
National Ethics Committee	16
Department of Health	2
International European institution	3
*Language*	
Just national language	18
National language and English translation	10
Just English	14

The most represented are UK (7 published ethical guidelines), France (6 published guidelines) and Spain (4 published guideline), followed by Switzerland, Germany (3 published guidelines) and Italy (2 ethical guidelines). The majority of the ethical guidelines was published by Scientific Society (21 ethical guidelines) and National Ethic Committees (16 ethical guidelines). 14 ethical guidelines were published in English and 10 ethical guidelines present the English translations and could be included in our analysis. We then excluded an ethical guideline because it presented an updated version.

Finally, twenty-two ethical guidelines met our inclusion criteria and were analyzed by argument-based analysis (as reported in Table 1).

As a result of the analysis and synthesis of the 22 individual guidelines, we ultimately identified the following ethical concepts and their related arguments, as described in Table 3:

1. Equity principle and emerging ethical theories.
2. Triage criteria.
3. Respecting patient dignity.
4. Decision making and quality of care.

**Table 3:** Identification of the ethical concept, their related arguments and the reference’ s guideline

Ethical Concepts	Related Arguments	Guidelines
Equity principle and emerging ethical theories	The egalitarian approach: equity and non discrimination	10, 11, 20–31
	The utilitarian approach: equity and the best use of resources	5, 30, 32–39
	The relationship between the equity principle and the Ethics of care framework	21, 22, 24, 25, 26, 27–31, 33, 34, 37–39
Triage criteria	Quantitative health-related triage criteria	28–30, 32, 33, 35–37, 39
	Patient-related clinical judgment and ‘questionable criteria’	10, 21, 22, 24–28, 30, 31, 33, 38
	Ethically unacceptable criteria and controversial application	11, 22, 24, 26, 28–30, 32, 33, 35, 39
Respecting Patient dignity	Palliative care	11, 24, 26, 28, 30, 33–37
	Considering patient’s will and wishes	28, 29, 32–36
	Individualized patient’s care	10, 11, 21, 25, 26, 27, 28, 31–32, 34, 36, 38
Decision making and quality of care	Ethical aspect of communication and triage management	11, 22, 25, 26, 34, 36–39
	Need of ethical support	21, 25, 28–30, 32, 34, 36, 37

### Equity principle and the emerging ethical theories

The analysis of the ethical guidelines highlights the central role of equity principle showing different meanings based on its theoretical relationship with three main ethical theories: egalitarianism, utilitarianism, and ethics of care.

#### The egalitarian approach: equity and non-discrimination

Inspired by the egalitarian approach, 14 ethical guidelines balance the principle of equity intended as equal treatment for equal needs with the principle of equality, that is equal treatment regardless of needs. The guidelines describe equality in terms of the respect for the absolute value of life and dignity of the person, as defined by the human rights framework [10, 11, 20–27]. As a result of this approach, triage criteria should be applied to every patient without discrimination or distinction among COVID and non-COVID patients [10, 11, 24–26, 28–31]. Following this perspective, some guidelines apply the “first come, first served” approach to choose between patients with the same urgency and needs [29, 30], even if it cannot be considered as the only criterion for allocation resources.

#### The utilitarian approach: equity and the best use of resources

10 guidelines balance the principle of equity with the principle of utility, which requires first to identify the type of outcomes that will be counted as improvements to the greatest common benefit. Utility principle is widely described as maximizing the benefits from scarce resources for the greatest number of people. It also consists of the “best value use” of resources [32–34] by reducing mortality and incrementing benefits in the society [5, 35–38]. Following the utilitarian approach, the total number of lives saved [32], the number of years of life saved [36] or the total number of years saved in relation to the quality of life and the capacity of the patient to benefit from the treatment are crucial aspects [30, 32–34, 37, 39]. The Italian ethical guidelines developed by the *Italian Society of Anesthesia Analgesia Resuscitation and Intensive Care* (*SIAARTI*) highlight that the “extraordinary nature of the situation” can justify the clinicians’ judgment about the inappropriate access of a patient to intensive treatment, namely to the pursuit of the greatest common benefit [36]. The Estonian ethical guidelines developed by the *University of Tartu* underlines that the patient-centered approach should be replaced with the community-centered approach. Consequently, physicians are not obliged to provide intensive care to a patient with a negative prognosis if that care is needed for the treatment of a patient with a better prognosis [32].

#### The relationship between equity principle and the ethics of care framework

15 guidelines balance the Beauchamp and Childress’ four principles of medical ethics [25, 27, 31–33], proportionality and appropriateness of care [24, 38, 39], and the health care professional’s responsibility to care [34]. The emerging meaning of equity in allocation resources lies in the concept of decisions taken “case by case”, without automatisms or criteria according to which the sick person would be excluded based on belonging to a category established a priori [22, 37] and providing assistance based on the needs of the individual patient, the realistic goal of care and the will of the individual concerned [21, 24–26, 28, 38]. This approach can be related to the ethics of care theoretical framework. It recognizes the value of each personal experience but also that human beings are interdependent [40, 41]. According to ethics of care, every moral choice or ethical issue is conceived as inserted in the relationship of care [42].

### Triage criteria

To apply the allocation resources in a practical situation, the guidelines identify a set of “triage criteria”, which identify who and how patients should access or be prioritized to intensive care. The most favorable prognosis is widely considered the main criterion to prioritize patients.[43] According with the equity principle discussed above, this criterion is defined by 2 different approaches: quantitative health-related triage criteria and patient-related clinical judgment.

#### Quantitative health-related triage criteria

The ethical guidelines here described define the most favorable prognosis in the patient's expected treatment outcomes in terms of duration and quality of life after intensive treatment [32, 33, 35, 37, 39]. The presence of life-short chronic diseases, severe comorbidities and the disease severity are also central criteria for non admission to ICU, and they are usually assessed by score system tools (e.g., the Clinical Frailty Score) [28–30, 32, 33, 35–37].

Some guidelines include age as a possible criterion. For example, two guidelines specify that triage criteria should also be based on the appraisal of the total number of lives saved, life years saved and patient’s years of life [35, 36]. One guideline specifies the possibility to introduce an age threshold to a patient’s access to intensive care due to the extraordinary situation [36], while others specify that age cannot be considered as a criterion in itself but in relation to the current clinical evaluation [29, 33].

Following this approach and the quantity of life-related criteria, some guidelines define those categories of people who do not access treatment: the terminally ill [29], those who do not consent to treatments through Advance Directives [29, 32, 35, 36] or Advance Care Planning [28, 29, 33], and patients with specific clinical diagnoses, such as dementia or cognitive impairments [29]. In this regard, other guidelines specify that the lack of ability to give consent and disability should not be considered a discriminatory factor to access to treatment [28]. One guideline specifies that priority should be a guarantee to a specific group, namely, people at risk and people with essential responsibilities and roles in the pandemic management, due to the principle of reciprocity [35].

The majority of guidelines do not expressly distinguish between withholding and withdrawing treatments. However, according to two guidelines, the physician has the right to withdraw the ICU resources from a patient with a negative prognosis if they are needed for the treatment of a patient with a better prognosis [32, 37]. This practice is linked with the regular assessment of patients: in facts daily re-evaluation of ICU patients is required by all the guidelines to determine whether criteria to undergo intensive treatment are still met.

#### Patient-related clinical judgment and “questionable criteria”

A number of ethical guidelines do not apply specific health-related clinical triage criteria but adopts a more general patient-related clinical judgment [10, 21, 24–27, 30, 31, 38]. In particular, the guideline developed by the *French national Consultative Ethics Committee* openly affirms that criteria are always questionable [21], and the *German Ethics Committee* affirms that any criteria should not overcome the human rights framework [22]. Following this approach, the triage criteria are defined by a case-by-case assessment of the patient’s clinical condition [25] and should take into consideration the urgency, the severity of comorbidity, the proportionality and appropriateness of the invasive treatments, and the treatment’s prognostic efficacy in terms of probable healing [24–26, 28–30]. Timely identification of disproportionate care is also required [29], and there is not an obligation to provide treatment which is not clinically appropriate [38]. In this regard, futility, proportionality or disproportionate care emerged among the ethical guidelines as criteria for withholding treatments and redirecting the therapeutic goal towards palliative care, only when ICU treatments are considered no more beneficial for the patient him/herself.

Moreover the health care professionals’ duty of care, which is linked to the ethics of solidarity between them and members of society [44], is also considered a relevant aspect to apply triage criteria in such a situation. Ethical guidelines underline clinicians’ responsibility to timely inform patients and their families about triage criteria and, principally, the responsibility to promote a discussion about the patient’s access to intensive care with timely advanced care planning [28–30, 33].

#### Ethically unacceptable criteria and controversial application

The majority of the guidelines report a set of ethically unacceptable criteria, including gender, ethnicity, sexual orientation, religion and political vision. They are justified on the basis of the equality principle with respect to human dignity and the human rights framework [22, 24, 28, 30, 32, 35]. Most of the guidelines place specific attention on age, which cannot be assumed as the only criterion [26, 29, 33], and disability [24, 28, 30, 39]; these groups are considered vulnerable people, and specific attention and particular effort should be made to ensure them equal rights and treatments [11].

Despite this, it is important to point out that there is controversy on the age issue. For example, the Swiss ethical guideline, while declaring to avoid any discrimination based on age, uses the age limit as an exclusion criteria in certain situation [33]. The Belgian document states that age in isolation cannot be used for triage decisions, but should be integrated with other clinical parameters: frailty and reduced cognition. They are specifically described as ‘independent predictors of outcome when elderly patients are admitted to the ICU’, but, as mentioned above, they would be effective only in combination with age [29].

### Respecting patient dignity

Respecting human dignity is a central aspect of the analyzed guidelines.

While the term ‘dignity’ is cited in each guideline, no explicit description of its meaning is provided. However, the general significance of dignity arose, and it can be described in terms of respecting the intrinsic value of each human beings (equality) while differentiating resources among people with different needs (equity) (Table 4).

**Table 4:** Emerging meaning of dignity among ethical guidelines

Country	Guidelines’ title	Mention of dignity	Appropriateness and proportionality	Equality	Equity	Emerging meaning of dignity
Austria	Management of scarce resources in healthcare in the context of the COVID-19 pandemic	The protection of the individuals and their *dignity* provided for in these fundamental rights implies the duty to provide healthcare to every person regardless of who they are, in other words, without distinction following non-medical criteria	There is no right to medical treatment that is not or no longer medically indicated	Equality is considered as a binding fundamental right Everyone has a right to life (Article 2 ECHR) and other relevant Fundamental rights in the medical context, such as in particular the right to respect for private life (Article 8 ECHR). (…) There is no justification for excluding a person from treatment based on criteria such as their remaining lifetime or quality of life. At the same time, it needs to be emphasized that there is no right to medical treatment that is not or no longer medically indicated	Equity: Some people are in need of special support to be able to effectively exercise their fundamental right to life and the access to associated medically indicated treatment, e.g. if they have a physical or mental/cognitive impairment. Such cases require not only the same, but possibly more resources to ensure that they have the same chance as people without such impairments	Respecting the intrinsic value of human beings—no discrimination (equality) while differentiate resources among people with different needs (equity)
Belgium	Ethical principles concerning proportionality of critical care during the COVID-19 pandemic: advice by the Belgian Society of IC medicine		Disproportionate care should be defined on a scientifically funded estimate of the expected outcome, which implies knowledge of an advanced care plan, the medical condition of the patient, the antecedents, the acute evolution of his condition, and a funded estimate of his prognosis with and without intensive care	In addition, non-COVID-19 patients should be evaluated according to the same criteria in order to avoid discrimination between both groups. Although an increased age is associated with worse outcomes in COViD-19, age in isolation cannot be used for triage decisions, but should be integrated with other clinical parameters. Frailty and reduced cognition, more than age, are independent predictors of outcome when elderly patients are admitted to the ICU		Dignity as respect of patient’s autonomy and patient’s choices and respecting the intrinsic value of human beings—no discrimination (equality)
Council of Europe	COMMITTEE ON BIOETHICS (DH-BIO) DH-BIO Statement on human rights considerations relevant to the COVID-19 pandemic	It is essential that decisions and practices meet the fundamental requirement of respect for human dignity and that human rights are upheld			The principle of equity of access to health care laid down in Article 3 of the Oviedo Convention must be upheld, even in a context of scarce resources. It requires that access to existing resources be guided by medical criteria, to ensure namely that vulnerabilities do not lead to discrimination in the access to healthcare. This is certainly relevant for the care of COVID-19 patients, but also for any other type of care potentially made more difficult with confinement measures and the reallocation of medical resources to fight the pandemic. The protection of the most vulnerable people in this context is indeed at stake, such as persons with disabilities, older persons, refugees and migrants. This concerns decisions to allocate scarce resources, to provide necessary assistance to those most in need	Respecting the intrinsic value of human beings—no discrimination (equality) while differentiate resources among people with different needs—vulnerable people (equity)
Estonia	Recommendations on clinical ethics for Estonian hospitals for distribution of limited health care resources during the COVID-19 pandemic	Beneficence, patients’ autonomy (including informed consent) and the principle of human dignity continue to be in effect		Equal treatment. The medicine system treats all patients equally regardless whether they have COVID-19 infection or some other severe illness Earlier arriving for treatment does not give any patient any advantage compared to those who come later		Respecting the intrinsic value of human beings—no discrimination (equality)
EGE	Statement on European Solidarity and the Protection of Fundamental Rights in the COVID-19 Pandemic	The protection of human health is accorded a much higher priority in the system of values of the European Union than economic interests. EU member states should jointly pursue the protection of health of EU citizens				Protection of Human health and human rights
France	COVID-19 Contribution from the French national Consultative Ethics Committee: Ethical issues in the face of a pandemic	A person's dignity does not depend on his or her usefulness. Thus, in a situation of scarcity of resources, medical choices, always difficult, have to be guided by ethical reflection that takes into account respect for the dignity of persons and the principle of fairness			The need for triage of patients raises a major ethical question of distributive justice, which in this case may lead to a differential treatment for patients infected with COVID-19 and those with other diseases. Those choices must always be explained and respect the principles of human dignity and fairness. It will also be necessary to be vigilant about the continuity of care for other patients	Differentiate resources among people with different needs (equity)
Germany	Solidarity and Responsibility during the Coronavirus Crisis	The guaranteeing of human dignity necessitates egalitarian equality and thus provides for corresponding basic protection for all against discrimination				Respecting the intrinsic value of human beings—no discrimination (equality) while differentiate resources among people with different needs (equity)
Ireland	Ethical Framework for Decision-Making in a Pandemic			The principle of fairness means that everyone matters equally, and under normal circumstances all individuals have an equal claim to healthcare		Respecting the intrinsic value of human beings—no discrimination (equality)
Italy	Clinical ethics recommendations for admission to intensive care and for withdrawing treatment in exceptional conditions of imbalance between needs and availble resources		All access to intensive care must be considered and communicated as an “ICU trial” only and therefore undergo daily reassessment of its appropriateness, based on goals of care and proportionality of care		It may be necessary to establish an age limit for admission to the ICU. It is not a question of making choices *merely according to worth*, but to reserve resources that could become extremely scarce to those who, in the first instance, have a greater likelihood of surviving and who, secondarily, will have more years of life saved, with a view to maximizing the benefits for the greatest number of people	Differentiate resources among people with different needs—vulnerable people (equity)
Republic of San Marino	Statement on ethical issues regarding to the use of invasive assisted ventilation in patient s all age with serious disabilities in relation to Covid-19 pandemic	Respect for human dignity is concretized allowing each person to experience a good death, through the precious tool of Palliative Care, which guarantee the control of pain and suffering, in the deep awareness that a person's life seriously ill and incurable, it never loses its intrinsic value nor the right to be supported and protected, therefore it reiterates that equal dignity must also be guaranteed to "non-treatable" victims, through taking charge and any sedation of pain		The founding principles of the Convention can be briefly indicated in equality and non discrimination and in the equality of opportunity (…) Equality of opportunity concerns the recognition of the right of access to goods and services, primarily health-related services		Respecting the intrinsic value of human beings—no discrimination (equality) Respecting dignity means offer patients good death and no suffer
Portugal	CNECV statement: Covid-19 key consideration	The protection of life, dignity and integrity of citizens is an ethical responsibility that involves political authorities at different levels, namely in the preparation of health and sanitary responses, while planning and organising access to healthcare				Respecting dignity as ethical responsibility to Respecting the intrinsic value of human beings—no discrimination (equality)
	Public health emergency situation due to the COVID-19 pandemic- Relevant ethical aspects	Duty to protect human health should be given precedence when confronted with possible economic interests			Care teams are responsible for assessing the clinical needs of each patient, namely their severity and urgency, and weighing the response according to the principle of equitable distribution of available resources, which, in a context of scarcity, is a highly demanding responsibility	Differentiate resources among people with different needs—vulnerable people (equity)
Spain	Report of the Ministry of Health on ethical aspects in pandemic situations: SARS-CoV-2	The very foundations of our rule of law, in particular our recognition of the equal intrinsic dignity of every human being		Accepting discrimination of this kind would mean giving less value to certain human lives due to their life-cycle stage, contradicting the very foundations of our rule of law, in particular our recognition of the equal intrinsic dignity of every human being	It will be necessary to combine the general framework for such criteria with a thorough reflection on the situation and circumstances of each particular patient, and assessing-within that general framework of guiding principles—the uniqueness and individuality of each person affected	Respecting the intrinsic value of human beings—no discrimination (equality) differentiate resources among people with different needs—vulnerable people (equity) Individualized care
Switzerland	Pandemic Covid-19: triage of intensive care treatments in case of scarcity of resources Indications for the implementation of chapter 9.3 of the directives of the ASSM "Measures of intensive care" (2013), updated version of March 24, 2020				Equity: Available resources are to be allocated without discrimination—i.e. without unjustified unequal treatment on grounds of age, sex, residence, nationality, religious affiliation, social or insurance status, or chronic disability	Respecting the intrinsic value of human beings—no discrimination
The Holy See	Pandemic and Universal Brotherhood	Decisions cannot be based on differences in the value of a human life and the dignity of every person, which are always equal and priceless			The decision concerns rather the use of treatments in the best possible way on the basis of the needs of the patient, that is, the severity of his or her disease and need for care, and the evaluation of the clinical benefits that treatment can produce, based on his or her prognosis. Age cannot be considered the only, and automatic, criterion governing choice. Doing so could lead to a discriminatory attitude toward the elderly and the very weak	Respecting the intrinsic value of human beings—no discrimination
UNESCO International Bioethics Committee (IBC)and theUNESCO World Commission on the Ethics of Scientific Knowledge and Technology (COMEST)	Statement on COVID-19: Ethical considerations from a global perspective	Procedures need to be transparent and should respect human dignity. Ethical principles enshrined in the human rights framework recognize the protection of health as a right of each human being				Respecting the intrinsic value of human beings—no discrimination; human right to health care
UK	Guidance: Responding to COVID-19: the ethical framework for adult social care	*Respect* This principle is defined as recognising that every person and their human rights, personal choices, safety and dignity matters	(Inclusiveness): consider any disproportionate impacts of a decision on particular people or groups	*Inclusiveness* This principle is defined as ensuring that people are given a fair opportunity to understand situations, be included in decisions that affect them, and offer their views and challenge. In turn, decisions and actions should aim to minimise inequalities as much as possible		Dignity as respect of patient’s autonomy and patient’s choices and respecting the intrinsic value of human beings—no discrimination (equality)
	Ethical considerations in responding to the COVID-19 pandemic	People should be treated as moral equals, worthy of respect				Respecting the intrinsic value of human beings—no discrimination
	COVID-19—ethical issues. A guidance note			*Equal respect* everyone matters and everyone matters equally, but this does not mean that everyone will be treated the same	*Fairness* everyone matters equally. People with an equal chance of benefiting from a resource should have an equal chance of receiving it—although it is not unfair to ask people to wait if they could get the same benefit later	Respecting the intrinsic value of human beings—no discrimination (equality) while differentiate resources among people with different needs (equity)
	Ethical dimension of COVID-19 for front-line staff		Front-line staff, policymakers, management and government have a responsibility to patients to ensure that any system used to assess patients for escalation or de-escalation of care does not disadvantage any one group disproportionately. Treatment should be provided, irrespective of the individual’s background (eg disability), where it is considered that it will help the patient survive and not harm their long-term health and wellbeing			Respecting people while differentiate resources among people with different needs (equity)
	Covid-19 Guidance: Ethical Advice and Support Framework	Respect All patients should be offered good quality and compassionate care Fairness Patients should be treated as individuals, and not discriminated against	Minimising harm Where there is a decision that a treatment is not clinically appropriate there is not an obligation to provide it, but the reasons should be explained to the patient and other options explored^8^ No active steps should be taken to shorten or end the life of an individual^9^, however the appropriate clinical decision may be to withdraw life prolonging or life sustaining treatment, or change management to deliver end of life care	It is important that patients are treated independent of suspected or confirmed COVID-19 status, and that any clinical decision guidance applies equally to all patients. The interests of each person are the concern of all of us, and of society ∙ The harm that might be suffered by every person matters, and so minimising the harm that a pandemic might cause is a central concern^10^		Respecting the intrinsic value of human beings—no discrimination (equality) while differentiate resources among people with different needs (equity)
	Coronavirus: Your frequently asked questions	If a decision is taken not to start or to withdraw some forms of treatment from a patient, doctors should still take all possible steps to alleviate the patient’s symptoms and distress and respect their dignity. The patient’s wishes, preferences and fears in relation to their future treatment and care should be explored as far as possible		Decisions are based on clinical need and the likely effectiveness of treatments, and don’t unfairly discriminate against particular groups		Respecting the intrinsic value of human beings—no discrimination (equality) while differentiate resources among people with different needs (equity). Respecting dignity means to offer patients good death and no suffer. Dignity as respect of patient’s autonomy

Moreover, ethical guidelines describe how to respect dignity through the application of palliative care, consideration of patients’ wills, and an individualized patient care.

#### Palliative care

Most of the guidelines include the provision of palliative care for all patients who will not receive ICU treatments. Two ethical guidelines explicitly argue that palliative care represents an approach to respect human dignity: people affected by a serious illness does not lose their intrinsic value as a person nor the right to be supported and protected [24, 34]. Patients who do not meet the triage criteria and cannot accede to intensive treatments, patients at the end of life, or patients who refuse the intervention should all be referred to palliative care, which aims to ensure a death without suffering and proximity to the family, despite the difficulties imposed by the situation [24, 26, 28, 34–37]. Some guidelines emphasize that patients who do not accede to the intensive treatment (or the ones whose treatments should be withdrawn) have the right to receive the best available alternative and compassionate care, that lies in the principle of continuity of care [11, 28, 30, 33, 37], and is supported by the principle of justice [35].

#### Considering the patient’s will and wishes

Another emerging way to respect patient dignity is dealing with patient’s will. A significant number of ethical guidelines include that physicians should take into consideration patient’s Advance Directives and his/her consent to intensive treatment or other treatment. Knowing the patient’s will and discussing the possibility of invasive treatment are associated with respect for the patient’s autonomy and moral values underlined by the 4 principles of medical ethics [28, 29, 32, 33]. The patient’s will represents a tool to help clinicians to make triage decisions and should be carefully evaluated in advance [29, 32, 34–36].

#### Individualized patient care

Some ethical guidelines highlight the necessity to provide individualized and person-centered care, even in the context of a public health emergency. This approach requires paying attention to vulnerabilities and to differentiating treatment considering the individual needs of each patient as the optimal way to avoid discrimination in access to health care [10, 21, 25, 26, 28, 30]. It develops the consideration that all patients should be offered good quality and compassionate care and treated as individuals, while recognizing that there may be a competing obligation to the wider population [11, 27, 38]. According to this approach, ICU treatments would be interrupted or not initiated only if they are considered not beneficial for the individual patient.

The communicative aspects of care and its implication on patients and familiars are also important for strengthening the care relationship [31, 32, 36, 38]. As specifically noted in the ethical guidelines provided by the *General Medical Council*, the health care professions should communicate openly and honestly with patients and the rest of the health care team about the decision-making process and the criteria for establishing priorities in individual cases [34].

### Decision making and quality of care

The decision-making process for allocation of resources is a frequent element of all guidelines. It is also built on ethical principles and represents an important aspect to ensure high-quality care and to strengthen the health care relationship, even in the urgency context. The institution of ethics support and ethics committee (or any other form of support for health care professionals) are also frequently required.

#### Ethical aspects of communication and triage management

Transparency, reasonableness, inclusiveness, openness and the uniform application of triage criteria at the different levels of care are important reference principles to ensure the non-arbitrariness of the final decision [11, 22, 25, 26, 37]. Procedures for decision making should respect human dignity [11], and clinicians should act with honesty and integrity [38]. The involvement of health care professionals not directly involved in the provision of intensive care is also underlined to mitigate the negative effects of pressure on doctors and teams [25], and a collective decision made by an ad hoc medical committee is often required [34, 36, 39]. An on-going revision of the guidelines, triage criteria and decisions is also a reiterated aspect to ensure that the best possible treatments are always guaranteed to each patient [36, 37].

#### Need of ethical support

Since these are very difficult decisions, the presence of local ethics committees, ethicists, or any other form of support, including psychological, aimed at managing and reducing the moral distress of health care professionals [21, 25, 28–30, 32, 34, 36, 37], is considered as an important aid for distributing and strengthening their sense of responsibility, applying ethical guidelines in their daily practice and, finally, making difficult decisions and facing direct dilemmas, especially in those extremely complex situations where suspension of already initiated treatments is required. Ethical guidelines have also noted that the application of a structured decision-making process and the identification of the persons responsible for the decision are fundamental to guarantee the patient, his family members and the community itself the trust pact and alliance on which the relationship of care is based and that the current emergency could dramatically corrupt [21].

## Discussion

The purpose of our study was to analyze how the European countries dealt with the allocation of scarce clinical resources at the beginning of the COVID-19 pandemic. We analyzed the ethical concepts and their practical consequences described in the ethical guidelines developed under COVID-19 by different institutional bodies (such as national ethics committees and health departments) among the European countries.

Our results are in line with similar articles comparing ethical guidelines developed under COVID-19 [43, 45, 46]. Differently from them, our analysis compared an higher number of guidelines and emphasized all the relevant ethical approaches, such as ethics of care and individualized patient care.

Each guideline analyzed recognizes respect for each human life's intrinsic value and the value of health as a human right as milestones to implement respect for human dignity.

Specifically, the emerging meaning of dignity is the intrinsic value of all the individuals who share the essential properties of human beings (“intrinsic dignity”). In recognition of that value, the health care professionals have a moral duty towards those who are suffering from disease and injury [47]. According with Jobgsen et al., the reference to equality and equity principle represents an area of consensus among European guidelines, and consequently the respect for human dignity lies in the principle of equal worth of people [45].

Nevertheless, during a pandemic, also the value of maximizing benefit emerged as an area of consensus [45]. Our results highlighted an implicit tension between respect for the equal right to health and risk of taking triage decision, including potential discriminatory criteria (such as patient’s frailty, short-term prognosis and cognitive impairment, which are linked to age). Then, the risk of applying a strictly utilitarian approach emerged, even if it was not a predominant approach for allocating resources among European guidelines [43]. This utilitarian consideration is most important, according to other non-European ethical guidelines, and in particular with American [9, 13] and Australian [48] guidelines. As noted by Emanuel et al., priority for limited resources should aim “both at saving the most lives and at maximizing improvements in individuals’ post treatment length of life”, which is “consistent both with utilitarian ethical perspective, focused on population outcomes, and with non-utilitarian views which emphasize the paramount value of each human life” [9]. The patient’s benefit and post-treatment outcomes are frequently cited in the European guidelines. It is often supported [5, 49, 50] the duty to care as the responsibility to respect the rights of patients to autonomy, transparency, privacy, and confidentiality of personal informations. These guidelines also require that procedures for taking informed consent and advance directives shall be observed and, where appropriate, legally authorized substitute decision-makers shall be consulted. Different from our results, the last guideline provided by *The Hastings Center* [51] affirms that an ethical allocation resource strategy first requires the protection of health care workers delivering care in the midst of the crisis, for without them and their extraordinary efforts, the entire health system would collapse. Subsequently, decisions about who receives treatment must center on prevention of SARS-CoV-2 transmission (public health), protection of individuals at highest risk, meeting societal needs, and promoting social justice [51]. Consequently, groups at highest risk, such as older adults, people with compromised immune systems, and people with underlying conditions (such as heart or lung diseases or diabetes) are another priority, as they are most likely to become seriously ill and die. This is in line with the concept of taking care of vulnerable groups, which was also underlined in our results.

Previous studies [52, 53] related to end-of –life care practices in ICU, highlight that decisions on withholding and withdrawing intensive treatments are mostly affected by patient age, acute and chronic diagnosis, number of days in ICU and cultural and religious beliefs [53]. Only 4 [28, 29, 35, 36] guidelines on COVID-19 pandemic are in line with those results, while a high number of guidelines underlined the importance of the individualized approach. It is essential to mention the difference between an acute and chronic diagnosis and COVID-19 illness. While in the first case can be clear what is overtreatment and the meaning of deterioration, during COVID-19 rapid deterioration does not necessary correspond to end of life, and thracheostomy can save the patient’s life. Consequently, short terms survival should be considered cautiously because it is not yet well known in Covid-19 [45].

Our results emphasize the need to respect the patient’s choices and will through advanced discussion on the access to intensive care and open and transparent communication about triage criteria. This reflects the attempt of health care professionals in non-pandemic situations to take individualized decision on the access to ICU, accordingly with clinical judgment and patient’s goals of care [54]. The importance of patient’s advance directives and patient’s will and the explicit recommendation to offer palliative care to patients whose treatment is withdrawn or withheld represent a consensus area among European ethical guideline [43, 45].

Despite our results recognize the value of both personalized care and relationship of care, many concerns regarding the effective possibility to respect patient’s consent and will arise in clinical practice, above all in pandemic situations: as noted by Angelos P et al., as scarcities increase, clinicians will increasingly be in a position in which they cannot respect all of their patient’s wishes, leading them to assume a paternalistic approach [55].

According to this study, the discussion of prognosis is central to obtaining informed consent for intubation, but, as noted by Zareifopoulos et al., in the absence of definitive data, it is not clear exactly what this discussion should entail [56]. Moreover, one of our guidelines affirms that the clinician’s judgment of inappropriateness would be justified by the critical situation’s extraordinariness, which reflects a paternalistic approach. This is in line also with Emanuel et al., who confirm that in a critical situation “the decision to withdraw a scarce resource to save others is not an act of killing and does not require the patient’s consent” [9].

According with similar studies [43, 45], our results show that the European ethical guidelines are very sensitive to the risk of discrimination arising from strict triage criteria, and particularly regarding age and disability. The difficult choices regarding admission to ICU of patients with advanced disease or elderly patients with multiple comorbidities was considered a ‘grey zone’ also in non-pandemic situations [54]. Discrimination based on age correlates the maximum benefit obtainable with the prioritization of younger people over old people, who have less life expectancy (both in terms of quantity and quality) and have already lived much more than young people [57]. This is widely spread among the American and Mexican ethical guidelines [58]. Particularly, White and Lo, 2020 justify “ageism” on the number of year saved [59], while the American Society of Geriatrics (ASG) assesses that rationing strategies that are based in part on age cutoffs could lead to persistent beliefs that older adults’ lives are less valuable than others’ lives or are even expendable [23].

Fighting the ageistic approach, Cesari and al. 2020 replaced the age criterion for the allocation of resources with a parameter more robust than age but equally easy to obtain and that can be used for critical and rapid decision-making, namely, the careful evaluation of the presence of comorbidity and functional status in addition to age. This is also marked by Auriemma et al. 2020 [59], who argue that an optimal policy for critical care resource allocation should not use categorical exclusions, in order to mitigate discrimination: ‘the feasibility virtues offered by such coarse systems are readily outweighed by their threats to justice, public trust, and clinician morale’. When the decision of using a ventilator for a person with respiratory distress is based on his/her birth date or other categorical exclusion criteria “we must realize that modern medicine may be at risk of having lost the meaning and value of the human life” [60].

Compared to European concerns, in the United States, the discussion on resource allocation and triage criteria dealt more deeply with issues of potential unjust discrimination for specific citizen group (namely elderly and disables). It  led to an open discussion and public engagement process to ensure equal access to health care that failed among European countries [46]. European ethical guidelines have been developed mainly by professionals societies or national bodies, resulting in a total lack of public involvement in identifying and discussing the principles that could guide scarce resources allocation.

Our results mention the importance of trust communication among health care professionals and patients and their familiars. The inclusion of a patient’s representative in developing ethical guidelines could improve health care professionals to make well-founded decisions in the interest of their patients.

As noted by Mannelli, the novelty of the current emergency has to do with the extraordinarily high number of people who find themselves personally affected by the implications of scarce resources allocation and who suddenly realize that the principle of “equals should be treated equally” may no longer be applicable [2].

## Conclusion

According to the ethical guidelines developed at the beginning of the COVID-19 pandemic, promoting how to figure out a way to personalize care during COVID-19 still represents a moral duty [5]. Being guided by the ethical reflection that considers respect for persons' dignity and the principle of equity, the difficult choices regarding patient prioritization and allocation of scarce resources should avoid unfair discrimination. At the same time, this kind of ethical reflection guarantees a relational approach to ethics, which includes appropriate and proportionate care, transparency and trust communication and, mostly, considering interconnection, vulnerability and shared humanity [44]. Personalized care in such a critical situation develops concretely by (a) a multidisciplinary, multidimensional and individualized evaluation of each patient; (b) the contextualization of ethical guidelines, involving who is directly called to their application; (c) an effective palliative care approach; and (d) the implementation of clinical ethics support, which represents a very important resource to help health care professionals in making difficult decisions [61].

Further studies can investigate the practical consequences of the application of the guidelines described in terms of quality of care and health care professionals’ moral distress.

## Supplementary Information

**Additional file 1: Appendix 1**. European ethical guidelines developed at the beginning of Covid-19 pandemic.

**Additional file 2: Appendix 2**. Conceptual scheme example.

## Data Availability

All data generated or analysed during this study are included in this published article (and its supplementary information files).

## Abbreviations

SARS-CoV-2: Severe acute respiratory syndrome coronavirus 2
COVID-19: COronaVirus Disease 2019
ICU: Intensive Care Unit
WHO: World Health Organization
QUAGOL: Qualitative Analysis Guide of Leuven
SIAARTI: Italian Society of Anesthesia Analgesia Resuscitation and Intensive Care
ASG: American Society of Geriatrics
