# Supplementary material for: Among equity and dignity: an argument-based review of European ethical guidelines under COVID-19
Source: BMC Med Ethics. 2021 Mar 31;22:36. doi: 10.1186/s12910-021-00603-9 (PMC8011067; doi:10.1186/s12910-021-00603-9)
Supplement: Supplementary file 1 — Additional file 1: Appendix 1. European ethical guidelines developed at the beginning of Covid-19 pandemic. [file 12910_2021_603_MOESM1_ESM.docx]

**Appendix 1-European ethical guidelines developed at the beginning of COVID_19 pandemic**

This appendix collects the websites we used to collect the ethical guidelines. We firstly searched ethical guidelines from National Ethics Committee’s *Publications* (Section I), we also investigated European National Bodies and Professional Associations’ recommendations (Section II) and finally we checked European International Organization’s website (Section III) .

**Section I**

1. <https://www.bundeskanzleramt.gv.at/en/topics/bioethics-commission/publications-bioethics.html>, Bioethics commission, Management of scarce resources in healthcare in the context of the COVID-19 pandemic, (German and English), *Geschäftsstelle der Bioethikkommission,* **Austria** (first version March 2020)
2. <https://www.ccne-ethique.fr/en/actualites/contribution-french-national-consultative-ethics-committee-covid-19-crisis-ethical-issues>, [COVID-19 Contribution from the French National Consultative Ethics Committee: Ethical issues in the face of a pandemic](https://www.ccne-ethique.fr/sites/default/files/publications/ccne_contribution_march_13_2020.pdf) (French and English), *The French National Ethics Council,* **France** (first version 13.3.2020)
3. <https://www.ccne-ethique.fr/fr/actualites/bulletin-ndeg1-le-ccne-instance-de-veille-ethique-dans-le-contexte-exceptionnel-de-la> [Réflexions et points d'alerte sur les enjeux d'éthique du numérique en situation de crise sanitaire aiguë (bulletin de veille n°1) (French)](https://www.ccne-ethique.fr/sites/default/files/publications/bulletin-1-ethique-du-numerique-covid19-2020-04-07.pdf) **France** (first version 7.4.2020)
4. <https://etene.fi/documents/1429646/21643455/Social-+och+h%C3%A4lsov%C3%A5rdsomr%C3%A5dets+etiska+principer+g%C3%A4ller+ocks%C3%A5+under+undantagsf%C3%B6rh%C3%A5llanden/615ad5af-e795-37ec-5d65-7225ec4fe605/Social-+och+h%C3%A4lsov%C3%A5rdsomr%C3%A5dets+etiska+principer+g%C3%A4ller+ocks%C3%A5+under+undantagsf%C3%B6rh%C3%A5llanden.pdf> [Riksomfattande etiska delegationen inom social- och hälsovården ETENE (Swedish)](https://etene.fi/documents/1429646/21643455/Social-+och+h%C3%A4lsov%C3%A5rdsomr%C3%A5dets+etiska+principer+g%C3%A4ller+ocks%C3%A5+under+undantagsf%C3%B6rh%C3%A5llanden/615ad5af-e795-37ec-5d65-7225ec4fe605/Social-+och+h%C3%A4lsov%C3%A5rdsomr%C3%A5dets+etiska+principer+g%C3%A4ller+ocks%C3%A5+under+undantagsf%C3%B6rh%C3%A5llanden.pdf) *National Advisory Board on Social Welfare and Health Care Ethics (ETENE)***, Finland.** (first verion 30.3.2020)
5. <https://www.ethikrat.org/publikationen/publikationsdetail/?tx_wwt3shop_detail%5Bproduct%5D=135&tx_wwt3shop_detail%5Baction%5D=index&tx_wwt3shop_detail%5Bcontroller%5D=Products&cHash=a37377aedcc6b8b131fce9a9146f9095> [Solidarity and responsibility during the coronavirus crisis](https://www.ethikrat.org/en/press-releases/2020/solidarity-and-responsibility-during-the-coronavirus-crisis/?cookieLevel=accept-all&cHash=7fd9da91d2848d9074e5f1cf0a7e9aa8) (German and english) *Deutscher Ethikrat* **Germany (first version 27.3.2020)**
6. <http://www.bioethics.gr/index.php/en/anakoinosis/2664-new-recommendation-of-the-hnbc-the-bioethical-dimension-of-individual-responsibility-in-response-to-covid-19-coronavirus> [The bioethical dimension of individual responsibility in response to COVID 19 (coronavirus)](http://www.bioethics.gr/images/pdf/GNOMES/Recommendation_coronavirus_FINAL_GR.pdf) (greek) *Hellenic National Bioethics Commission,* **Greece (not mentioned)**
7. <https://www.gov.ie/en/publication/dbf3fb-ethical-framework-for-decision-making-in-a-pandemic/> Ethical framework for decision-making in a pandemic, (English) *Department of Health* **Irland (First version 24.4.2020)**
8. <http://bioetica.governo.it/italiano/documenti/pareri-e-risposte/covid-19-la-decisione-clinica-in-condizioni-di-carenza-di-risorse-e-il-criterio-del-triage-in-emergenza-pandemica/> [COVID-19: La decisione clinica in condizioni di carenza di risorse e il criterio del "triage in emergenza pandemica" (Italian)](http://bioetica.governo.it/media/3987/p136_2020_covid-19-la-decisione-clinica-in-condizioni-di-carenza-di-risorse-e-il-criterio-del-triage-in-emergenza-pandemica.pdf), *Comitato Nazionale per la Bioetica (CNB)* **Italy(first version)**
9. <https://cne.public.lu/dam-assets/fr/publications/avis/Prise-de-position-COVID-19.pdf>

[Repères éthiques essentiels lors de l’orientation des patients dans un contexte de limitation des ressources thérapeutiques disponibles due à la crise pandémique du COVID-19](https://cne.public.lu/dam-assets/fr/publications/avis/Prise-de-position-COVID-19.pdf) (French), *Luxembourgish National Ethics Committee***, Luxembourg. (First version 31.3.2020)**

1. [https://www.who.int/ethics/topics/outbreaks-emergencies/Pos-CNECV-Covid-19-EN.pdf?ua=1Position of the National Council of Ethics for the Life Sciences: Public Health Emergency Situation Due to the COVID-19 Pandemic - Relevant Ethical Aspects](https://www.who.int/ethics/topics/outbreaks-emergencies/Pos-CNECV-Covid-19-EN.pdf?ua=1) **(**English, portugese**), Portugal. (First version April 2020)**
2. <https://www.who.int/ethics/topics/outbreaks-emergencies/Statement-CNECV-Covid19_Key-Considerations.pdf?ua=1>-[CNECV Statement: COVID-19 Key Considerations](https://www.who.int/ethics/topics/outbreaks-emergencies/Statement-CNECV-Covid19_Key-Considerations.pdf?ua=1) *National Ethics Council for the Life Sciences* **(**English**) Portugal (First version April 2020)**
3. <http://www.sanita.sm/on-line/home/bioetica/comitato-sammarinese-di-bioetica/pareri-csb.html>[Statement on ethical issues regarding the use of invasive assisted ventilation in patients all age with serious disabilities in realation to the COVID-19 pandemic](http://www.sanita.sm/on-line/home/bioetica/comitato-sammarinese-di-bioetica/documents-in-english/documento2116023.html) (English, Italian) *National Bioethics Committee* **Republic of San Marino (First version March 2020)**
4. <https://www.gov.si/assets/ministrstva/MZ/DOKUMENTI/KME/Stalisce-KME-o-odlocanju-zdravnikov-v-zvezi-z-vkljucevanjem-respiratorjev-v-zdravljenje-hudo-prizadetih-bolnikov-z-boleznijo-povzroceno-z-virusom-SARS-CoV2.pdf>[KME's position on physicians' decision-making regarding the involvement of respirators in the treatment of severely affected patients with SARS CoV2 disease (Slovene)](https://www.gov.si/assets/ministrstva/MZ/DOKUMENTI/KME/Stalisce-KME-o-odlocanju-zdravnikov-v-zvezi-z-vkljucevanjem-respiratorjev-v-zdravljenje-hudo-prizadetih-bolnikov-z-boleznijo-povzroceno-z-virusom-SARS-CoV2.pdf) *Republic of Slovenia National Medical Ethics Committee (KME)* **Slovenia (First version 24.3.2020)**
5. <http://www.comitedebioetica.es/documentacion/#doc_crisis_coronavirus>

[Informe del Comité de Bioética de España sobre los aspectos bioéticos de la priorización de recursos sanitarios en el contexto de la crisis del coronavirus](http://assets.comitedebioetica.es/files/documentacion/Informe%20CBE-%20Priorizacion%20de%20recursos%20sanitarios-coronavirus%20CBE.pdf), (spanish) *Comité de Bioética de España* **Spain (Fist version 25.3.2020)**

1. <https://www.nek-cne.admin.ch/inhalte/Medienmitteilungen/de/Medienmitteilung_NEK_Pandemie_D.pdf> [Corona-Pandemie: Schutz des Lebens und Solidarität stehen aus ethischer Sicht im Zentrum](https://www.nek-cne.admin.ch/inhalte/Medienmitteilungen/de/Medienmitteilung_NEK_Pandemie_D.pdf) (German) *Nationale Ethikkommission im Bereich der Humanmedizin* **Switzerland (First version 27.3.2020)**
2. <http://www.academyforlife.va/content/pav/it/notizie/2020/pandemia-e-fraternita-universale.html>[Pandemic and Universal Brotherhood: Note on the Covid-19 emergency](http://www.academyforlife.va/content/dam/pav/documenti%20pdf/2020/Nota%20Covid19/Note%20on%20the%20Covid-19%20emergency_ENG_.pdf) (English, Itaiano, spagnolo , francese, giapponese) *Pontifical Academy for Life* **The Holy See:**
3. <https://www.nuffieldbioethics.org/news/statement-covid-19-and-the-basics-of-democratic-governance> [COVID-19 and the basics of democratic governance](https://www.nuffieldbioethics.org/news/statement-covid-19-and-the-basics-of-democratic-governance), (English) *The Nuffield Council on Bioethics* **The United Kingdom (First version 25.3.2020)**
4. <https://www.nuffieldbioethics.org/publications/covid-19> [Ethical considerations in responding to the COVID-19 pandemic](https://www.nuffieldbioethics.org/assets/pdfs/Ethical-considerations-in-responding-to-the-COVID-19-pandemic.pdf) (English) *The Nuffield Council on Bioethics* **The United Kingdom (First version 17.3.2020)**

**Section II**

1. <https://www.hartcentrumhasselt.be/professioneel/nieuws-professioneel/ethical-principles-concerning-proportionality-of-critical-care-during-the-covid-19-pandemic-advice-by-the-belgian-society-of-ic-medicine> Ethical principles concerning proportionality of critical care during the 2020 COVID-19 pandemic in Belgium, (English), [*Belgian Society of Intensive Care Medicine* **Belgium**](http://www.siz.be/wp-content/uploads/COVID_19_ethical_E_rev3.pdf) (First version 26.3.2020)
2. <https://www.regionaalhaigla.ee/sites/default/files/documents/Estonian_recommendations_COVID19_eng.pdf>Recommendations on clinical ethics for Estonian hospitals for distribution of limited health care resources during the COVID-19 pandemic (English) **Estonia (First version 8.4.2020)**
3. <https://www.conseil-national.medecin.fr/publications/actualites/decisions-medicales-contexte-crise-sanitaire-dexception>[, Décisions médicales dans un contexte de crise sanitaire et d'exception (French)](https://www.conseil-national.medecin.fr/publications/actualites/decisions-medicales-contexte-crise-sanitaire-dexception) Conseil National de l‘Ordre des Médecins **France (First version march 2020)**
4. <http://www.sfap.org/document/enjeux-ethiques> Enjeux éthiques de l’accès aux soins de réanimation et autres soins critiques (SC) en contexte de pandémie COVID-19 (French), [*La Société Française d'Accompagnement et de soins Palliatifs (SFAP)*:](http://www.sfap.org/system/files/gt_etic_rea_covid_16_mar_20_19h.pdf)  **France First version March 2020)**
5. <https://www.coreb.infectiologie.com/UserFiles/File/procedures/rpmo-ethique-rea-covid-19-vf-24-corr26-mar20-2.pdf>, [Aspects éthiques et stratégiques de l’accès aux soins de réanimation et autres soins critiques (SC) en contexte de pandémie COVID-19 (French)](https://www.coreb.infectiologie.com/UserFiles/File/procedures/rpmo-ethique-rea-covid-19-vf-24-corr26-mar20-2.pdf), Recommandation professionnelle multi-disciplinaire opérationnelle (RPMO): **France (First version 24.3.2020)**
6. [<https://sfar.org/download/decision-dadmission-des-patients-en-unites-de-reanimation-et-%20unites-de-soins-critiques-dans-un-contexte-depidemie-a-covid-%2019/?wpdmdl=25432&refresh=5e7b782d19bb81585149997> Décision d’admission des patients en unités de réanimation et unités de soins critiques dans un contexte d’épidémie à Covid-19 (French)](https://sfar.org/download/decision-dadmission-des-patients-en-unites-de-reanimation-et-%20unites-de-soins-critiques-dans-un-contexte-depidemie-a-covid-%2019/?wpdmdl=25432&refresh=5e7b782d19bb81585149997) *Société Française d’Anesthésie-Réanimation*, **France (First version 19.3.2020)**
7. <https://www.divi.de/empfehlungen/publikationen/covid-19/1549-entscheidungen-ueber-die-zuteilung-intensivmedizinischer-ressourcen-im-kontext-der-covid-19-pandemie-klinisch-ethische-empfehlungen/file> Entscheidungen über die Zuteilung intensivmedizinischer Ressourcen im Kontext der COVID-19-Pandemie Version 2 . 16-4-2020,(German)  *Der Deutschen Interdisziplinären Vereinigung für Intensiv- und Notfallmedizin (DIVI),* **Germany (Second version 16.4.2020)**
8. <https://www.divi.de/empfehlungen/publikationen/covid-19/1540-covid-19-ethik-empfehlung-v2/file> [Entscheidungen über die Zuteilung von Ressourcen in der Notfallund der Intensivmedizin im Kontext der COVID-19-Pandemie](https://www.divi.de/empfehlungen/publikationen/covid-19/1540-covid-19-ethik-empfehlung-v2/file) 25-3-2020, (German) *Der Deutschen Interdisziplinären Vereinigung für Intensiv- und Notfallmedizin (DIVI),* **Germany (First version 25.3.2020)**
9. <http://www.siaarti.it/SiteAssets/News/COVID19%20-%20documenti%20SIAARTI/SIAARTI%20-%20Covid-19%20-%20Clinical%20Ethics%20Reccomendations.pdf> [*Italian Society for Anesthesia, Analgesia, Resuscitation & Intensive Care (SIAARTI)*  Clinical Ethics Recommendations for the Allocation of Intensive Care Treatments in exceptional, resource-limited circumstances](http://www.siaarti.it/SiteAssets/News/COVID19%20-%20documenti%20SIAARTI/SIAARTI%20-%20Covid-19%20-%20Clinical%20Ethics%20Reccomendations.pdf) (Italian, english, german) **Italy (First version 6.3.2020)**
10. <https://static1.squarespace.com/static/5dfb95f97d6e9d050dba42f3/t/5e7f7209abb56b3bf6dafc7a/1585410574353/Norway_Covid-19+priority+guidelines.pdf>, [Helsedirektoratet: Prioritering av helsehjelp i Norge under covid-19 pandemien (Norwegian)](https://static1.squarespace.com/static/5dfb95f97d6e9d050dba42f3/t/5e7f7209abb56b3bf6dafc7a/1585410574353/Norway_Covid-19+priority+guidelines.pdf) **Norway (First version 25.3.2020)**
11. <https://rm.coe.int/pandemic-covid-19-spain-eng/16809e3a78>, Ministry of Health Report on Ethical Issues in Pandemic Situations: SARS-CoV-2 *,(*Englis*h, Spanish) Ministry of health***, Spain (First version 3.4.2020)**
12. <https://www.medintensiva.org/es-recomendaciones-eticas-toma-decisiones-dificiles-avance-S0210569120301108> Recomendaciones éticas para la toma de decisiones difíciles en las unidades de cuidados intensivos ante la situación excepcional de crisis por la pandemia por COVID-19: revisión rápida y consenso de espertos (Spanish), *Sociedad Española de Medicina Intensiva, Crítica y Unidades Coronarias (SEMICYUC),* **Spain (First version 15.4.2020)**
13. <http://www.bioeticayderecho.ub.edu/sites/default/files/doc_recom-pandemia.pdf> R[ecomendaciones para la toma de decisiones éticas sobre el acceso de pacientes a unidades de cuidados especiales en situaciones de pandemia](http://www.bioeticayderecho.ub.edu/es/recomendaciones-toma-decisiones-eticas-sobre-acceso-pacientes-unidades-cuidado) (Catalan) , *Observatorio de Bioética y Derecho (OBD)*, **Spain (First version March 2020)**
14. <https://www.socialstyrelsen.se/globalassets/sharepoint-dokument/dokument-webb/ovrigt/triage-arbetssatt-vardcentral-covid19.pdf> Kunskapscentrum Katastrofmedicin & Karolinska Institutet (on behalf of the National Board of Health & Welfare): Triage/flöden och arbetssätt vid covid-19 (Swedish ), *National Board of Health & Welfare,* **Sweden (First version 7.4.2020)**
15. <https://www.sams.ch/en/Ethics/Topics-A-to-Z/Intensive-care-medicine/Triage-in-case-of-bottlenecks-chronology.html>,  [COVID-19 pandemic: triage for intensive-care treatment under resource scarcity](https://www.samw.ch/dam/jcr:c1f2b1d3-95d4-486a-8c59-e5668e74e97b/guidelines_v2_sams_triage_intensive_care_resource_scarcity_20200324.pdf) – Version I, 20 March 2020, (Englis, French, German, Italian), *Swiss Academy of Medical Sciences*, Switzerland (First version 20.3.2020)
16. <https://www.sams.ch/en/Ethics/Topics-A-to-Z/Intensive-care-medicine.html>, [*Swiss Academies of Arts and Sciences & Swiss Society for Intensive Care Medicine (SGI)*: Intensive care medicine: triage in case of bottlenecks](https://www.samw.ch/en/Ethics/Topics-A-to-Z/Intensive-care-medicine.html), Version II, 24 March 2020 (English, French, German and Italian) **Switzerland (Second version 24.3.2020)**
17. <https://www.bma.org.uk/media/2360/bma-covid-19-ethics-guidance-april-2020.pdf>

[COVID-19 – ethical issues. A guidance note](https://www.bma.org.uk/media/2226/bma-covid-19-ethics-guidance.pdf). (English) *British Medical Association*, **The United Kingdom, (Frst version April 2020)**

1. <https://www.nhshealthatwork.co.uk/images/library/files/Clinical%20excellence/Ethical_guidance_for_publication_V1_final.pdf>  [Ethical dimensions of COVID-19 for front-line staff](https://www.rcplondon.ac.uk/file/20551/download), *Royal College of Physicians,*(English) **The United Kingdom (First version 31.3.2020)**
2. [<https://www.gov.uk/government/publications/covid-19-ethical-framework-for-adult-social-care/responding-to-covid-19-the-ethical-framework-for-adult-social-care>, Responding to COVID-19: the ethical framework for adult social care](https://www.gov.uk/government/publications/covid-19-ethical-framework-for-adult-social-care/responding-to-covid-19-the-ethical-framework-for-adult-social-care) (English) *Department of Health & Social Care* , **The United Kingdom (First version 19.3.2020)**
3. <https://www.gov.scot/publications/coronavirus-covid-19-ethical-advice-and-support-framework/>, [COVID-19 Guidance: Ethical Advice and Support Framework](https://www.gov.scot/binaries/content/documents/govscot/publications/advice-and-guidance/2020/04/coronavirus-covid-19-ethical-advice-and-support-framework/documents/covid-19-cmo-ethical-advice-and-standards-3-april-2020/covid-19-cmo-ethical-advice-and-standards-3-april-2020/govscot%3Adocument/COVID-19%2BCMO%2Bethical%2Badvice%2Bsupport%2Bguidance%2B-%2Bv2.2%2B-%2B3%2BApril%2B2020.pdf) (English), *Scottish Government*, **The United Kingdom (First version 3.4.2020)**
4. <https://www.gmc-uk.org/ethical-guidance/ethical-hub/covid-19-questions-and-answers#Decision-making-and-consent>, Coronavirus: Your frequently asked questions (English), *General Medical Council*, **The United Kingdom (not mentioned)**

**Section III**

1. <https://rm.coe.int/inf-2020-2-statement-covid19-e/16809e2785> COMMITTEE ON BIOETHICS (DH-BIO) DH-BIO Statement on human rights considerations relevant to the COVID-19 pandemic (English), **Council of Europe (First version 14.4.2020)**
2. <https://unesdoc.unesco.org/ark:/48223/pf0000373115> STATEMENT ON COVID-19: ETHICAL CONSIDERATIONS FROM A GLOBAL PERSPECTIVE Statement of the UNESCO International Bioethics Committee (IBC) and the UNESCO World Commission on the Ethics of Scientific Knowledge and Technology (COMEST **IBC (International Bioethics Commitee) and**[**World Commission on the Ethics of Scientific Knowledge and Technology**](https://unesdoc.unesco.org/query?q=Corporate:%20%22IBC%22&sf=sf:*) **(Comest) (First version 6.4.2020)**
3. <https://ec.europa.eu/info/sites/info/files/research_and_innovation/ege/ec_rtd_ege-statement-covid-19.pdf> Statement on European Solidarity and the Protection of Fundamental Rights in the COVID-19 Pandemic **EGE (European Group on Ethics in Science and New Technologies) (First version 2.4.2020).**
