# Supplementary material for: Among equity and dignity: an argument-based review of European ethical guidelines under COVID-19
Source: BMC Med Ethics. 2021 Mar 31;22:36. doi: 10.1186/s12910-021-00603-9 (PMC8011067; doi:10.1186/s12910-021-00603-9)
Supplement: Supplementary file 2 — Additional file 2: Appendix 2. Conceptual scheme example. [file 12910_2021_603_MOESM2_ESM.docx]

**Appendix 2- Conceptual scheme example**

This Table represents an example of how ethical guidelines were conceptualized into conceptual scheme. In this case, we provided the conceptual scheme of the Italian ethical guideline, *Clinical Ethics Recommendations for the Allocation of Intensive Care Treatments in exceptional, resource-limited circumstances* elaborated by the [Italian Society for Anesthesia, Analgesia, Resuscitation & Intensive Care (SIAARTI)](http://www.siaarti.it/SiteAssets/News/COVID19%20-%20documenti%20SIAARTI/SIAARTI%20-%20Covid-19%20-%20Clinical%20Ethics%20Reccomendations.pdf)

| Ethical Framework | **Clinical appropriateness and proportionality**  **Distributive justice and appropriate allocation of scarce resources** |
| --- | --- |
| Access criteria to ICU treatments | they should be flexible and can be adapted locally according to the availability of resources. They apply to all intensive care patients, not only to those who have been infected by COVID-19. )  . |
|  | Possible age limit: priorization of those have a greater likelihood of surviving and who, secondarily, will have more years of life saved, with a view to maximizing the benefits for the greatest number of people |
|  | In addition to age, the presence of comorbidities and functional status must be carefully evaluated. |
|  | The possible existence of prior expressed wishes (Advance Directives) |
|  | A judgment of inappropriateness due to the extraordinary nature of the situation (an extreme imbalance between demand and availability). |
| Decision-making process | Shared decision-making among health care professionals |
|  | Advance individualized discussion on the criteria for admission to the ICU for every patient ( list of patients) |
|  | daily reassessment of ICU treatments appropriateness, based on goals of care and proportionality of care |
| Practical consequencies | Provision of palliative care (also palliative sedation) for people excluded from ICU treatments. |
|  | Support Health care professionals to deal with such critical scenarios ( exchanging information among centers and individual professionals, monitoring of any possible professional burnout and moral distress). |
|  | Considering the consequences to the families of patients hospitalized in the ICU during COVID-19 |
